# Effect of short-term regulated temperature variations on the swimming economy of Atlantic salmon smolts

**DOI:** 10.1093/conphys/cox025

**Published:** 2017-05-04

**Authors:** C. M. Alexandre, A. P. Palstra

**Affiliations:** 1 MARE—Centro de Ciências do Mar e do Ambiente, Universidade de Évora, Largo dos Colegiais 2, 7004-516Évora, Portugal; 2 Wageningen Marine Research, Wageningen University and Research, Korringaweg 5, 4401 NT Yerseke, The Netherlands; 3 Wageningen Livestock Research, Animal Breeding and Genomics, Wageningen University and Research, PO Box 338, 6700 AH Wageningen, The Netherlands

**Keywords:** Hydropeaking, intermittent respirometry, oxygen consumption, *Salmo salar*, streamflow regulation

## Abstract

Migratory species travelling long distances between habitats to spawn or feed are well adapted to optimize their swimming economy. However, human activities, such as river regulation, represent potential threats to fish migration by changing environmental parameters that will have impact on their metabolism. The main objective of this study was to evaluate the changes in the swimming energetics of a salmonid species, Atlantic salmon (*Salmo salar* L.), caused by short-term temperature variations that usually result from the operation of hydroelectrical dams. Intermittent flow respirometry in swim tunnels allows to obtain high resolution data on oxygen consumption of swimming fish which can reflect aerobic and anaerobic metabolism. This method was used to compare the metabolic rates of oxygen consumption before, during and after sudden thermal change. Control (no temperature variation) and experimental (temperature variation of approximately 4°C in 1 h) swimming trials were conducted to achieve the following objectives: (i) quantify the variations in oxygen consumption associated with abrupt temperature decrease, and (ii) assess if the tested fish return quickly to initial oxygen consumption rates. Main results revealed that Atlantic salmon smolts show a strong response to sudden temperature variation, significantly reducing the oxygen consumption rate up to a seven-fold change. Fish quickly returned to initial swimming costs shortly after reestablishment of temperature values. Results from this study can be used to evaluate the species-specific effects of the applied operation modes by hydroelectrical dams and to increase the success of conservation and management actions directed to fish species inhabiting regulated rivers.

## Teaser

This study revealed that Atlantic salmon smolts respond to short-term water temperature drops, like changes occurring in rivers regulated for hydropower production, by significantly reducing their rate of oxygen consumption during swimming activity. Fish show resilience by quickly returning to initial swimming costs after reestablishment of the original temperature values.

## Introduction

Short-term streamflow regulation performed by dams operating for hydropower production (i.e. hydropeaking) is a drastic form of regulation in which high amplitude changes in flow occur suddenly and at completely unnatural rates. Within only a few hours the discharge can become many-fold higher and lower again (Poff *et al*., 1997; Bunn and Arthington, 2002). Besides streamflow, the abiotic component of riverine ecosystems considered to be most affected by hydropower dams is the thermal regime (Poff *et al*., 1997; Naiman *et al*., 2008). Usually, there are major shifts in thermal patterns downstream of dams because water is released from below the thermocline, which is often deoxygenated and considerably colder than the water that would be normally transported in the system (Olden and Naiman, 2010). Like streamflow, below these dams, riverine thermal variations usually occur abruptly and several times per day and specific components of the thermal regime (e.g. amplitude of variation, maxima and minima, etc.) also have relevance for the ecology and physiology of freshwater organisms (Poff *et al*., 1997). Therefore, the integrity of the thermal regime may be just as important as the integrity of the natural flow regime (Olden and Naiman, 2010) and should be given relevance and priority when studying the effects of hydropower production on aquatic organisms.

From the standpoint of ichthyofauna, the high and unpredictable environmental variability caused by hydropower generation is even more important than the simple alteration of flow magnitudes (Scruton *et al*., 2003; Vehanen *et al*., 2005). On one hand, ecological effects of hydropeaking on fish behaviour are well known (Taylor *et al*., 2014; Alexandre *et al*., 2015; Boavida *et al*., 2016) On the other hand, physiological effects (e.g. swimming demand, oxygen consumption and internal temperature regulation) caused by the increased environmental variability are, until this date, still poorly understood (Geist *et al*., 2005; Taylor *et al*., 2014). Moreover, specifically concerning the thermal regime, very few studies (Preece and Jones, 2002; Todd *et al*., 2005) have attempted to explore the individual and interactive effects of abrupt thermal modification related with dam operation on the riverine biota, especially on freshwater fish.

Considering the scarcity of information about hydropeaking effects on the biology and physiology of affected freshwater fish assemblages, this study marks one of the first attempts to study the swimming economy of a fish species when subjected to abruptly varying environmental factors, more specifically water temperature. We aimed to assess the changes in the oxygen consumption rate of Atlantic salmon (*Salmo salar* L.) smolts, in response to water temperature variations within the range that is usually associated to known hydropeaking phenomena. When studying smolts, knowledge on the relationship between fish movement and environmental variation during downstream migration is of high concern. Streamflow and temperature regimes are the two most important variables controlling migration of salmonid smolts to marine waters (Aldvén *et al*., 2015), therefore, significant alterations to these patterns downstream of hydropower dams, caused by hydropeaking operations, can severely impair this ecological process and affect juvenile Atlantic salmon's growth and survival.

Intermittent flow respirometry (Steffensen, 1989) was used to compare oxygen consumption rate (ṀO_2_) before, during and after abrupt temperature variation with the following specific objectives: (i) quantify the aerobic energy consumption variations associated with temperature decrease, and (ii) assess if fish can resume quickly previous physiological swimming costs. This method was consistently used in recent years to successfully assess the effect of natural long-term or circadian temperature fluctuations in the swimming performance and metabolism of distinct fish species (Beauregard *et al*., 2013; Chabot *et al*., 2016; Enders and Boisclair, 2016), but, to our knowledge, none addressed the effects of sudden and more accentuated temperature fluctuations usually associated to hydropeaking operations. This type of information assessed at a small physiological scale is almost always left out of hydropeaking-related studies which are normally more ecologically oriented, but can represent the basal explanation for some of the described responses at the assemblage level. Therefore, insights from this study can be crucial for the management and conservation of fish populations from rivers that undergo this type of flow regulation.

## Methods

Experimental protocols complied with the current laws of the Netherlands and were approved by the animal experimental committee (DEC nr. 2014064).

### Swimming trials and oxygen consumption rate measurements

Experiments to assess the effects of abrupt temperature variation on the metabolic costs of the swimming target species, the Atlantic salmon, were conducted at Wageningen Marine Research (Yerseke, The Netherlands) facilities during the spring of 2015, using four 127 L Blazka-type swim tunnels (van den Thillart *et al*., 2004 for a detailed description). Swim tunnels were placed in a climate room (15°C) with water recirculating from ambient tanks placed near the tunnels. Before each experiment, the water in the ambient tanks was renewed with fresh tap water. Each swim tunnel had a bypass system where a galvanic oxygen electrode, connected to a 4-channel respirometry system (DAQ-PAC-G4; Loligo Systems, Tjele, Denmark), was inserted to allow recording of dissolved oxygen concentration. During respirometry, water in the swim tunnels was recirculated through the bypasses and oxygen concentration in the system decreased due to the oxygen consumption of the swimming fish. Swimming trials were conducted with farmed Atlantic salmon smolts [*n* = 48; standard Body Length (BL): 10.30 ± 0.93 cm (mean ± standard deviation); Body Weight (BW): 19.78 ± 4.92 g] which were transported by truck from Norway to the IMARES facilities, under adequate conditions of temperature and oxygen, well in advance to the beginning of the experiments. Fish were housed in a 800 l tank with freshwater at a light regime of 14 h light:10 h dark, before and during the trial periods. Fish were fed twice per day *ad lib* with commercial feed pellets (crude protein 43%, ether extract 29%, ash 7%, 3 mm, Skretting), except when they resided in the swim tunnels.

We performed a total of four trials, using all four tunnels in each trial, to determine and compare Atlantic salmon's oxygen consumption rate when subjected to stable (two control trials) and varying (two treatment trials) temperature. The amplitude and rate of temperature variation used in these treatment trials were in accordance with the imposed photoperiod regime, and were based on the thermal regime registered downstream of a typical hydroelectrical dam from the Central Portugal, the Raiva dam, located in River Mondego (WGS84—Lat: 40°18′33″; Long: 8°14′59″), one of the largest and most important watercourses of the Iberian Peninsula. Temperature variation parameters downstream from this dam were previously assessed during a year using temperature data loggers. Information gathered during this period revealed that hydropeaking events in River Mondego caused a decrease in water temperature of ~4°C in approximately 1 h.

Before each swimming trial, 12 Atlantic salmons were anaesthetized by immersion in a bath with 2-phenoxyethanol at a concentration of 0.4 ml.L^−1^, measured (standard BL; ±1 mm), weighed (BW; ±0.1 g) and randomly distributed over the four swim tunnels. Considering potential biases that could result from using small fish in larger respirometer tunnels (Svendsen *et al*., 2016a,b), such as the one used in this study, we used three fish per tunnel to reduce the respirometer-to-fish volume ratio. Fish were left inside the tunnels, swimming in water at ambient temperature, until the next day (15 h), to acclimate to experimental conditions. Next day, each experiment was started exactly within the same hour period (9:00–10:00 a.m.) to avoid data biases related with circadian activity rhythms of the tested fish. In all trials fish were swimming below their critical swimming speed (Peake, 2008) at a moderate constant velocity of 4 BL. s^−1^ (based on the mean standard length of the three fish per swim tunnel with a range between 37.3–44.8 cm.s^−1^). The choice for the swimming velocity of 4 BL. s^−1^ was made on basis of a preliminary quick test in which this speed was determined as the lowest speed fully forcing fish to swim. Below 4 BL. s^−1^ fish could lay still on the bottom using the pectoral fins as anchors. The applied swimming speed of 4 BL. s^−1^ was close to the optimal swimming speed of 3.77 ± 0.10 BL.s^−1^ that was determined on fish from the same batch in the same set-up (Böhm and Palstra, unpublished data).

During any type of swimming trial, one of the most commonly observed effects is solid blocking (SBE), which should be considered and corrected if needed, particularly when the ratio between fish square area and swim tunnel cross-sectional area is above 10% (Bell and Terhune, 1970). For this study, we performed a calculation of the error associated to solid block effect (SBE) to assess the need of a posterior correction of swim speed values. For all our trials, the ratio between the square area of the three fish and cross area of the swim tunnel working section was approximately 1%, so the SBE can be considered negligible (Bell and Terhune, 1970). When calculated, the associated fractional error is *ca.* 0.008 and the solid blocking correction factor is estimated as *ca.* 0.1, which would not change significantly the swimming speeds used. Therefore, swimming speeds applied in this study were not corrected for SBE.

In the treatment trials, water temperature of 15°C was kept constant for a 1-h period, after which water in the ambient tank was rapidly decreased (1 h) within the range previously defined as related with hydropeaking events (4°C), until reaching 11°C. Fish were maintained swimming at this lower temperature for a period of 30 min, after which the temperature was again increased, during another hour period, until reaching the initial temperature of 15°C, mimicking what usually happens in the river/hydropower dam scenario from which the experimental pattern was derived. Sudden decrease and increase of water temperature was performed by using a set of two automatic chillers (HC-1000A, Valkenswaard, the Netherlands) and six submersible aquarium heaters, respectively. During the 2:30 h period of water temperature variation, this parameter was measured every 5 min in the ambient tank. Following this procedure, fish were kept swimming for three more hours, after which the trials were ended, resulting in a total trial duration of approximately 7 h. Oxygen content in the swim tunnels was continuously measured during the entire trial duration and so was the fish oxygen consumption, except for the flushing periods (i.e. 10 min) that were periodically performed to restore oxygen in the system to saturation levels before each new measuring period and to change temperature in the swim tunnel in accordance to the ambient tank. For the control trials, water temperature inside the ambient tank and swim tunnels was kept constant during the total duration of the experiment. We tried to maintain similar flushing periodicity between treatment and control trials but, since temperatures were kept constant in the latter, fewer periods were needed to measure oxygen values. The operator was the same for all the experiments and was constantly present to observe the behaviour of the swimming fish. The wall effect of the used swim tunnels comprises about two centimeters but fish were never observed to take advantage of it. At the end of the fish experiments, control and treatment blank trials (using a similar procedure but without fish swimming in the tunnels) were conducted to analyse, and if necessary compensate, for background oxygen consumption.

### Data analysis

No background consumption was detected in the blank tests, so we could directly calculate the rate of O_2_ consumption for each measuring period (and for all three smolts per tunnel) of each control and treatment trial (ṀO_2_, mg.kg^−1^.h^−1^) without any compensation, using the following expression:

$${ṀO}_{2}=\frac{{∆\mathrm{sat}}_{\left(h\right)}\times {\mathrm{mg}}_{O_{2}}}{{\mathrm{BW}}_{t}},$$

where ${∆\mathrm{sat}}_{\left(h\right)}$ is the proportion (%) of decline in oxygen saturation inside the swim tunnels per hour, ${\mathrm{mg}}_{O_{2}}$ is the amount of oxygen in mg per % saturation under each respective temperature level, and ${\mathrm{BW}}_{t}$ is the total fish body mass in each swim tunnel. The average (mean ± SD) *r*^2^ value associated with the slopes of O_2_ variation over time (Table 1), considering all trials, was 0.894 ± 0.069. ṀO_2_ calculations for each measuring period were performed and standardized for time periods with different durations throughout the trials (between 13 and 100 min). Non-linear oxygen depletion data during the mixing phase were omitted from analyses (Svendsen *et al*., 2016a).

**Table 1:** Biometrics of the Atlantic salmon smolts used in the respirometry trials

Fish ID	SL (cm)	BW (g)	*K*	Swim tunnel	Type of trial	*r*^2^ values (mean ± SD)
#1	10.5	20.6	1.78	1	Control	
#2	10.0	18.6	1.86			0.993 ± 0.003
#3	9.5	14.8	1.73			
#4	9.5	15.3	1.78	2	Control	
#5	10.7	19.3	1.57			0.973 ± 0.024
#6	9.0	12.4	1.70			
#7	10.0	16.6	1.66	3	Control	
#8	9.6	15.4	1.74			0.975 ± 0.016
#9	8.4	10.5	1.77			
#10	10.7	23.2	1.89	4	Control	
#11	8.5	11.1	1.81			0.993 ± 0.003
#12	10.4	20.8	1.85			
#13	10.8	22.5	1.79	1	Control	
#14	11.5	25.1	1.65			0.956 ± 0.030
#15	8.9	13.5	1.91			
#16	11.4	25.4	1.71	2	Control	
#17	11.3	21.5	1.49			0.956 ± 0.030
#18	8.9	11.5	1.63			
#19	11.0	20.4	1.53	3	Control	
#20	10.1	20.0	1.94			0.889 ± 0.196
#21	10.1	18.8	1.82			
#22	11.4	25.5	1.72	4	Control	
#23	11.4	23.5	1.59			0.889 ± 0.196
#24	9.8	15.5	1.65			
#25	11.1	24.6	1.80	1	Treatment	
#26	9.4	13.7	1.65			0.862 ± 0.102
#27	10.3	18.4	1.68			
#28	10.2	22.1	2.08	2	Treatment	
#29	10.5	21.4	1.85			0.862 ± 0.102
#30	8.4	10.7	1.81			
#31	10.5	21.5	1.86	3	Treatment	
#32	10.1	19.5	1.89			0.853 ± 0.100
#33	10.2	19.5	1.84			
#34	11.1	24.1	1.76	4	Treatment	
#35	10.4	18.5	1.64			0.852 ± 0.163
#36	9.4	14.3	1.72			
#37	11.9	32.4	1.92	1	Treatment	
#38	11.3	24.3	1.68			0.852 ± 0.153
#39	10.4	19.7	1.75			
#40	10.5	22.6	1.95	2	Treatment	
#41	11.3	26.7	1.85			0.862 ± 0.103
#42	11.0	24.2	1.82			
#43	11.2	24.8	1.76	3	Treatment	
#44	10.9	22.5	1.74			0.898 ± 0.090
#45	10.5	19.9	1.72			
#46	11.0	24.6	1.85	4	Treatment	
#47	10.9	21.8	1.68			0.891 ± 0.123
#48	8.1	11.7	2.21			

Average *r*^2^ values for slopes of O_2_ variation over time are also presented for each trial. SL—Standard length; BW—Body Weight; *K*—Fulton's Body Condition Factor.

Before any data analysis, assumptions for the use of appropriate parametric statistical methodologies, data normality and homogeneity of variances, were tested using the Shapiro–Wilk and Levene tests, respectively. Before the analysis of ṀO_2_ variation within each type of trials, univariate analyses of variance (ANOVA) were applied to check for differences in fish size (SL), fish weight (BW) and fish body condition (Fulton's Condition Index, $K=100\times \mathrm{BW}/{\mathrm{SL}}^{3}$) between control and treatment trials. After these preliminary analyses, and since multiple and consecutive O_2_ measurements were conducted for the same group of fish, a repeated-measures ANOVA was used to test for significant differences in fish ṀO_2_ between measuring periods within each trial, control and treatment, followed by Tukey post-hoc tests to identify significantly different periods.

## Results

Fish size (ANOVA, *F*_1,46_ = 1.25; *P >* 0.05), weight (ANOVA, *F*_1,46_*=* 3.45; *P >* 0.05) and Fulton's Body Condition Factor (ANOVA, *F*_1,46_*=* 3.83; *P >* 0.05) were not significantly different between control and experimental trials (Table 1), allowing us to continue with the planned ṀO_2_ comparisons without considering potential bias from these factors in the results.

**Figure 1:**
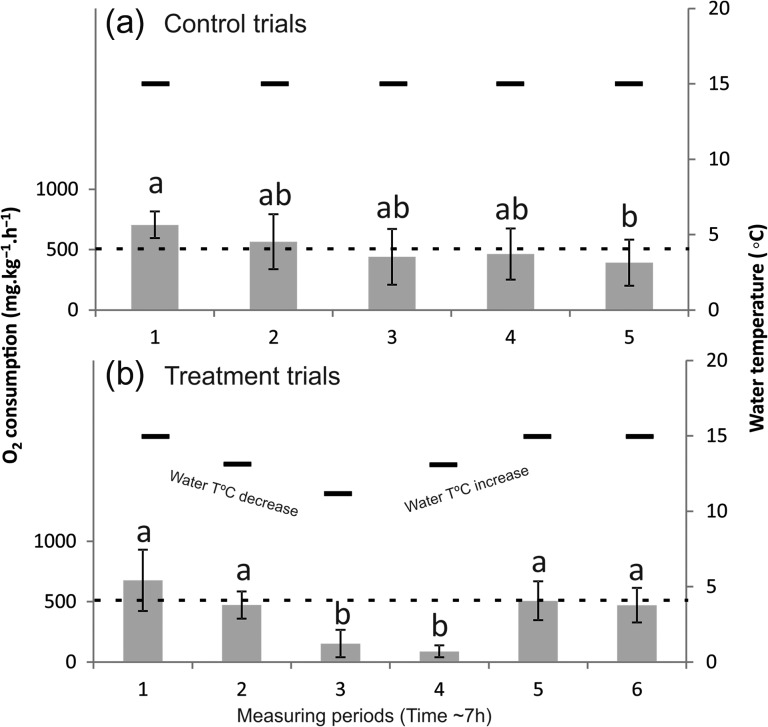
Average oxygen consumption (ṀO_2_ average ± SD; mg.kg^−1^.h^−1^) for each measuring period in control (**a**) and treatment (**b**) fish swimming trials; Distinct letters (a and b) above bars indicate significantly different measuring periods; Steps of temperature variation steps during the experiments are represented by the black short lines; Average ṀO_2_ calculated for the control trials, at 15°C (514 mg.kg^−1^.h^−1^) is represented by the black dashed line.

Atlantic salmon's ṀO_2_ showed significant differences between measuring periods during the control trials (Repeated-measures ANOVA, *F*_4,28_ = 4.41; *P* < 0.05). Temperature was kept constant during these trials but significant differences in ṀO_2_ existed between the first (Period 1) and last (Period 5) measuring period, as revealed by following Tukey post-hoc tests, with higher MO_2_ at the start (Fig. 1a). During the treatment trials, the pattern of ṀO_2_ by the tested Atlantic salmons was quite distinct from the controls, clearly responding to the temperature variation protocol (Fig. 1b). In this case, highly significant differences between the experimental measuring periods were identified (Repeated-measures ANOVA, *F*_5,35_ = 55.46; *P* < 0.001). More specifically, Tukey post-hoc tests revealed the existence of differences in Atlantic salmon's O_2_ consumption between the lower ṀO_2_ values during Periods 3 and 4, corresponding to the phases of sudden temperature variation, and the other measuring periods with significantly higher MO_2_ values. Average O_2_ consumption values registered during Periods 3 and 4 were clearly well below the average ṀO_2_ at the regular water temperature of 15°C (514 mg.kg^−1^.h^−1^). Oxygen consumption by Atlantic salmons during measuring Periods 5 and 6, recorded after the increase of water temperature back to initial values, was statistically similar (*P* > 0.05) to the first two measuring periods, recorded before the sudden decrease of water temperature.

## Discussion

### Metabolic responses to temperature changes

Many studies describe water temperature as the master variable controlling fishes’ swimming metabolism and energetic costs (Brett, 1965; Beauregard *et al*., 2013; Enders and Boisclair, 2016). General assumptions state that, when fish are exposed to temperature changes during a sufficiently long period, they can obtain optimal performance by altering either their behaviour (preference/avoidance) or their physiology (adaptation/acclimation) (Bernatchez and Dodson, 1985; Lee *et al*., 2003; Oligny-Hébert *et al*., 2015). However, certain short-term variations in temperature, such as the ones occurring periodically in rivers regulated for hydroelectric production, may be unavoidable and their effects on the swimming metabolism of fish are much less known than the impacts of long-term thermal changes. Usually, a decrease in temperature results in a decrease in the rates of enzymatic reactions resulting in a reduction in metabolic rate of oxygen consumption (Hochachka and Somero, 2001). This is a well-established assumption in the field of fish physiology and our data corroborate it. In the control trials, Atlantic salmon's ṀO_2_ remained stable except for a significant decrease after nearly 6–7 h of swimming, probably due to training and/or habituation (Beamish, 1978; Janz *et al*., 1991). In the treatment trials, MO_2_ followed the trend of environmental variation and significantly decreased (drop of *ca.* 70–80%) almost immediately after the sudden 4°C decrease in water temperature. After this, ṀO_2_ rose again to previous values when temperature resumed its initial value. The overall pattern observed in this study, in which higher ṀO_2_ values were associated to higher temperatures, and vice-versa, is consistent with previous studies for other fish species, namely rainbow trout (*Oncorhyncus mykiss*, Dickson and Kramer, 1971), sockeye salmon (*Oncorhynchus nerka*, Brett and Glass, 1973), largemouth-bass (*Micropterus salmoides*, Beamish, 1978; Cooke *et al*., 2001) and coho salmon (*Oncorhynchus kisutch*,  Lee *et al*., 2003). When studying Atlantic salmon with similar length as ours (*ca.* 10–15 cm) Enders *et al*. (2003) obtained ṀO_2_ values between 146 and 442 mg.kg^−1^.h^−1^ for fish swimming at 15°C, of which values for the smaller fish are comparable to the average ṀO_2_ value of 514 mg.kg^−1^.h^−1^ obtained in our study. Beauregard *et al*. (2013) and Oligny-Hébert *et al*. (2015), when studying Atlantic salmon juveniles, also reported a significant effect of diel temperature fluctuations (±2.5°C) on fish standard metabolic rate. Results from these studies indicate that circadian temperature fluctuations can incur an additional metabolic cost to salmons, consequently affecting their growth. Our study showed that hydropeaking-related temperature changes are also affecting salmons’ swimming metabolism. These type of artificial temperature changes occur more suddenly and at higher magnitudes than natural diel temperature variations. Therefore, effects of human-induce thermal regulation on fish bio-ecology, namely growth patterns, can also occur, perhaps at a larger extent. However, most of the existent studies report on swimming trials where fish were subjected to distinct experimental protocols (i.e. performing several trials at different temperatures rather than performing one trial with a sudden temperature variation, or inducing temperature changes of lower magnitude) making it difficult to compare results. Lee *et al*. (2003) also analysed the effect of temperature on swimming performance and oxygen consumption of two salmonid species (sockeye and coho salmon). These authors showed that temperature changes of approximately 5°C were responsible for a significant drop of routine ṀO_2_, about 50% of the initial ṀO_2_ value, corroborating the results obtained in the present study. Differences between our study and Lee *et al*. (2003) regarding the drop of oxygen consumption as a percentage of the initial values may reflect the type of protocol applied, since in the Lee *et al*. study, temperature variations were applied between and not within swim trials, probably reducing the degree of immediate impact on tested fish.

The results gathered for the second objective of this study, to assess the recovery of the target species after being subjected to short-term temperature changes, provide new and valuable insights about the way freshwater fish react to this phenomenon. After being subjected to a sudden 4°C decrease in water temperature, Atlantic salmons lowered ṀO_2_ values and rapidly re-established their regular metabolism when temperature returned to initial values. Earlier laboratory-based studies, also developed with hatchery-raised salmonid specimens (rainbow trout and sockeye salmon), show that these species can recover quickly when subjected to swimming speed tests. A 45-min recovery period was sufficient for these fishes to be able to repeat their previous swimming performance, both in terms of critical speed and ṀO_2_, even when they swam to exhaustion, which was not the case in our study (Jain *et al*., 1997; Farrell *et al*., 1998). As for the recovery period in our study, between the period of lower water temperature (Period 3) and the period in which temperature resumed its initial values (Period 5), ṀO_2_ remained low (*c.f.* Period 4 in Fig. 1b) although fish were swimming for an entire hour. If there was a ‘quasi’-linear relationship between temperature and ṀO_2_ (Hochachka and Somero, 2001), Periods 2 and 4 (both at *~*12°C) would be expected to be similar in terms of swimming costs but this was not the case in our study. During temperature rise, there is an apparent delay of its effect on Atlantic salmon’ ṀO_2_, but this delay seems to disappear when fish resume their initial ṀO_2_ level as soon as temperature is back to 15°C. More experiments are needed to support these data but, from our study, it can be concluded that typical scenarios of sudden thermal changes, like hydropeaking events, do not have a durable effect on swimming salmon smolts. As is shown by our results, after environmental conditions have returned to normal, fish regain rapidly previous metabolic rate levels. Absence of higher ṀO_2_ than the initial values after return may indicate the absence of stress induced by a short-term regulated temperature variation of 4°C but cortisol measurements may support this conclusion in future experiments.

### Conservation issues and future directions

The results obtained in this study can be the starting point for collection of a large set of physiological data that can be used to improve the management of hydroelectrical dams to promote more effective conservation and protection actions for affected fish species and populations. Migratory species such as the Atlantic salmon travel long distances between habitats to spawn or feed and are well adapted to optimize swimming economy (Lennox *et al*., 2016). Unexpected extreme values and short-term variations of either temperature or water velocity, such as the ones that usually happen in rivers regulated for hydropower production, can alter active metabolic rates and contribute to incomplete or delayed fish migrations through difficult stretches of impounded river systems (Macdonald *et al*., 2000). Knowledge about the physiological responses and recovery rates of fishes to sudden environmental changes is extremely important to promote timely migratory passages and reproduction success, when salmonids, as well as other migratory fish, face repetitive hydraulic and thermal challenges during their upstream migration in flow regulated rivers (Farrell *et al*., 2003).

In this study, we tested the effect of thermal variation but there are other biological (e.g. sex, energy reserves, gonad maturation) and environmental (e.g. dissolved oxygen, turbidity and current velocity) factors that may also influence oxygen consumption rates of individual migratory fish (Dickson and Kramer, 1971). Concerning specifically the described environmental factors, most of them can be severely and suddenly altered when hydropower dams operate. Future studies should evaluate the effects of these factors, individually and multifactorially, on the swimming economy of fish. Besides that, more insights should be gained on the species-specific effects of hydropeaking to increase the representativeness and applicability of obtained results and the suitability of conservation and management measures. Future work should address the effects of hydropeaking-related abiotic changes in other fish species, particularly cyprinids, which are abundant in world regions currently facing increasing hydroelectric exploitation (e.g. Europe and Australia; Arthington, 2012).
